# Intent to stay in the nursing profession and associated factors among nurses working in Amhara Regional State Referral Hospitals, Ethiopia

**DOI:** 10.1186/1472-6955-13-24

**Published:** 2014-08-25

**Authors:** Eshetu Haileselassie Engeda, Anteneh Messele Birhanu, Kefyalew Addis Alene

**Affiliations:** 1 Department of Nursing, College of Medicine and Health Sciences, University of Gondar, Gondar, Ethiopia; 2 Department of Public Health Officer, Institute of Public Health, College of Medicine and Health Sciences, University of Gondar, Gondar, Ethiopia

**Keywords:** Intent to stay, Nurse, Amhara region, Ethiopia

## Abstract

**Background:**

Nurses are essential to the health care delivery system especially to meet the health related millennium development goals. However, despite the significant shortage of nurses in Ethiopia, research in the country regarding nurses’ intent to stay in their profession is lacking. This study assessed intent to stay in the nursing profession and associated factors among nurses working in referral hospitals, Amhara Regional State, Ethiopia.

**Methods:**

Institution-based cross-sectional study was conducted among 389 nurses from April 8 to May 5, 2013. Stratified random sampling technique was used to select the study participants from five referral hospitals. Data were collected using pretested and structured self-administered questionnaires. Descriptive statistics were conducted to summarize the sample characteristics. Backward stepwise logistic regression model was fitted and adjusted odds ratio with 95% confidence interval was calculated to identify associated factors.

**Results:**

The proportion of nurses who reported intent to stay in the nursing profession was 39.8%. Age 40 to 49 (AOR [95% CI] 4.5 [1.6-12.8]), being married (AOR [95% CI] 2.0 [1.0-3.8]), having a bachelor degree in nursing (AOR [95% CI] 2.2 [1.2-4.1]), satisfaction with: autonomy and professional opportunities (AOR [95% CI] 2.6 [1.2-5.9]), scheduling (AOR [95% CI] 3.4 [1.6-7.5]), and pay and benefits (AOR [95% CI] 8.8 [4.5-17.1]); high continuance commitment (AOR [95% CI] 2.4 [1.3-4.8]) and high normative commitment (AOR [95% CI] 3.7 [1.9-7.2]) were the significant predictors of intent to stay in the nursing profession.

**Conclusions:**

Intent to stay in the nursing profession was low among nurses working in Amhara Regional State referral hospitals. Interventions aimed at increasing the professional autonomy of nurses and revising the current salary and other duty payments are vital.

## Background

Nurses, by delivering holistic patient care around the clock [1,2], are the keystones at each step in the health care delivery system [3], and their contribution is essential to meet the health related Millennium Development Goals [4]. However, the shortage of nurses has become a critical challenge across the globe [5], particularly in Sub-Saharan Africa. For instance, it was predicted that in 2015 the density of nurses and midwives in Sub-Saharan countries, including Ethiopia will be 0.65 per 1,000 population, which is substantially lower than the World Health Organization’s standard at 2.2 [6]. Consequently, failure to intervene on the nursing staff shortage at any level in the health system will compromise the quality of care [7].

Although there are many determinants related to the nursing shortage, high turnover is found to be the major contributor [8], which in turn is predominantly associated with nurses’ intent to stay in their profession [9,10]. Thus, the meaning of the nursing shortage includes not only the shortage of persons qualified in nursing but also the shortage of nurses who intend to stay in their profession [5]. Tourangeau and Cranley hypothesized the following six predictors of nurses’ intent to stay (ITS) in their profession: job satisfaction (JS), personal characteristics of nurses, work group cohesion and collaboration, manager ability and support, burnout, and organizational commitment (OC) of nurses. However, in their research only four were significantly associated with nurses’ ITS: job satisfaction, socio-demographic characteristics, work group cohesion and collaboration, and organizational commitment [11].

In a substantial number of research findings, most of the variance of ITS was consistently explained by JS. [9,12-14]. For instance, an Irish study identified JS as the most accurate predictor of ITS in that nurses who had high level of JS were more likely to show intent to stay [13]. Similarly, Chinese [15] and South African [16] studies showed the same results in that satisfied nurses were more likely to demonstrate ITS as compared with unsatisfied ones.

Organizational commitment was also among the major predictors of nurses’ ITS [17-19]. Tourangeau and Cranley defined OC as “the degree of attachment that nurses have towards their employer” (pp 501) [11]. In the three-component conceptualization model of Meyer and Allen, OC was further subdivided into three separate themes of affective commitment (AC), normative commitment (NC) and continuance commitment (CC) [20] and this model remains the leading approach in studying OC by different researchers [21]. In this model, individuals remain employed either because they are emotionally attached to the values and goals of the organization (AC), they feel they have an obligation (NC), or they are aware they would lose a lot if they left (CC). Therefore, OC serves as a stabilizer in strengthening employees’ behavioural intentions [19] and it plays a significant positive role in maintaining ITS [22]. Bakhshi et al. also concluded that the more committed individuals are the more likely they tend to stay in their organization [23].

Others have also found that certain socio-demographic characteristics were related to nurses’ ITS. In Montreal, Canada, about ten nurse personal characteristics were studied in relation to ITS. In this study, the findings revealed that marital status and educational level were significantly associated with ITS. Moreover, the survey showed that ITS was higher for nurses with diploma than nurses with baccalaureate degree [24]. In one Chinese study, bivariate analyses of nurses’ socio-demographic characteristics, including age, educational level, years of employment and job position, only age and job position were significantly and positively associated with ITS [14]. Another Chinese study strengthens these findings in that there were significant correlations between age and years of employment with ITS [15]. Similarly, a South African survey also supported this study in that increasing age was found to predict turnover intent. In the same study, educational level was found to be significantly and negatively associated with ITS in that higher educated nurses were more than twice as likely to consider leaving the profession [16].

Despite numerous studies in other countries, no studies on nurses’ intent to stay in the profession have been conducted in Ethiopia making it difficult to set appropriate nurse retention interventions and policies. Furthermore, because Ethiopia has a severe shortage of nurses overall, it is imperative to understand the factors associated with nurses’ intent to stay in the profession to prevent further reduction in the workforce. Therefore, this study was conducted in order to fill the knowledge gap regarding the factors associated with Ethiopian nurses’ ITS in the profession.

## Methods

An institution-based cross-sectional study was conducted from April 8 to May 5, 2013 at all 5 of the referral hospitals located in the regional state of Amhara, Northwest Ethiopia – Gondar University referral hospital, Felege Hiwot referral hospital, Debremarkos referral hospital, Debrebrhan referral hospital and Dessie referral hospital. Each hospital serves as a referral centre for more than five million people in its catchment area. Four of the hospitals are under the administration of Amhara Regional State health bureau and the other, Gondar University referral hospital is a university hospital under the administration of the Federal Ministry of Education.

The sampling frame for this study was employed nurses working in one of the 5 referral hospitals. Sample size was determined by using single population proportion formula by considering the following assumptions: 95% confidence interval (CI), 50% proportion (since there was no previous study in the study areas), 5% marginal error. By adding 10% non-response rate, the final sample size was 423. Stratified sampling technique was used to select the study participants. The assumption to use this method was the potential difference in organizational factors which could affect intent to stay in the nursing profession. The samples were proportionally allocated to each hospital. Finally respondents were selected using computer generated random number.

The primary outcome measure of interest was intent to stay in the nursing profession measured with a six item scale (α = 0.95) adopted from the Shanghai study [15]. The items were answered on a five-point Likert scale with response options ranging from 1 (strongly disagree) to 5 (strongly agree).

The independent variables included socio-demographic characteristics (age, gender, marital status, work experience as a nurse, job position, current area of practice, highest level of nursing education, having dependent family members and type of hospital), job-satisfaction and organizational commitment. Job satisfaction was measured with a thirty seven-item scale that included five subscales (adopted from previous study [25]): autonomy and professional opportunities, scheduling, support, relationship and interaction, and pay and benefits. These items were answered on a five-point Likert scale with response options ranging from 1 (very dissatisfied) to 5 (very satisfied). In the same manner, organizational commitment was measured with a twenty two-item scale that included 3 subscales (adopted from previous study [26]: affective commitment, continuance commitment and normative commitment. In the same fashion, the items were answered on a five-point Likert scale with response options ranging from 1 (strongly disagree) to 5 (strongly agree) [See Table 1 for the results of the Cronbach’s alpha].

**Table 1:** Description of scales of intent to stay, job-satisfaction and organizational commitment used by the current study

**Scale**	**Cronbach’s alpha**	**Number of items**
Intent to stay	0.95	6
Autonomy and professional opportunities	0.90	10
Scheduling	0.88	6
Support	0.89	7
Relationship and interaction	0.80	4
Pay and benefit	0.91	10
Affective commitment	0.74	8
Continuance commitment	0.75	8
Normative commitment	0.88	6

The data were collected using a pre-tested and structured English version self-administered questionnaire. Eight BSc holders for data collection and five graduating class MSc students (one for each hospital) for supervision were recruited and one day’s training was given. At each data collection site, the aim of the research was explained to the study subjects before they completed the questionnaire. Data quality was maintained by using the following methods. First, those questionnaires whose internal consistencies had been checked by previous researchers were used after carefully adopting them in to the current context and without changing the original meaning. Second, the adopted questionnaires were pre-tested on twenty two nurses who were working out of the study area and necessary amendment was made accordingly. Third, training was given for data collectors and supervisors. Fourth, supervision was done throughout the data collection period. Fifth, data were checked for consistency and completeness before entry to computer software for analysis. Finally, Epi Info software was used to control the potential errors associated with data entry.

The data were coded, entered into Epi Info 7, and exported to SPSS version 20 software for analysis. At the beginning of the analysis, summation of the scores for the scale, intent to stay and each of the subscales for job satisfaction and organizational commitment was made. Then, the variables were re-coded and dichotomized. Descriptive statistics were used to illustrate the means, standard deviations, medians and frequencies of the study variables. Bivariate analysis was computed and those variables whose p values less than or equal to 0.2 were fitted into the backward stepwise multivariate logistic regression model. Odds ratios with 95% confidence interval were used to determine the strength of association between dependent and independent variables. P-values less than or equal to 0.05 were considered as statistically significant.

Ethical clearance was obtained from Institutional Review Board of University of Gondar. Then official letter obtained from administrative body of each hospital. The purpose of study was well explained to the study participants and written consent was obtained. Confidentiality was maintained at all levels of the study by avoiding use of name and other identifiers. Participants’ involvement in the study was on voluntary basis; participant’s who were unwilling to participate in the study and those who wish to quit their participation were informed to do so without any restriction.

## Results

### Sample characteristics

Of the total 423 nurses contacted to participate in the study, 389 returned the questionnaire for a response rate of 91.9%. Approximately 53% of the participants were female, the majority were married (59.6%) and nearly 52% had a bachelor degree in nursing. The mean (SD) age was 32.94 (5.9) years. Most of the respondents were from referral hospitals under the administration of the regional health bureau (63.8%), and the greatest proportion of respondents worked in either surgical (27.5%) or outpatient departments (26.2%). The median (IQR) work experience as a nurse was 6 (4–9) years. Most of the respondents were staff nurses (90.5%) and the majority (55.3%) had no dependent family members living with them [Table 2].

**Table 2:** Socio-demographic characteristic of nurses in the 5 referral hospitals, Amhara regional state, Ethiopia, 2013

**Variable**	**Number**	**Percent**
**Sex**		
Male	182	46.8
Female	207	53.2
**Age**		
20-29	116	29.8
30-39	221	56.8
40-49	52	13.4
**Marital status**		
Unmarried^a^	157	40.4
Married	232	59.6
**Highest level of nursing education**		
Diploma (2 or 3 years)	188	48.3
Bachelor degree	201	51.7
**Type of hospital**		
Nurses from non-teaching		
referral hospitals	248	63.8
Nurses from a teaching		
referral hospital	141	36.2
**Work experience as a nurse**		
0-10 years	324	83.3
11-20 years	52	13.4
21-30 years	13	3.3
**Job position**		
Staff nurse	352	90.5
Head nurse	37	9.5
**Current area of practice**		
Medical department	99	25.4
Surgical department	107	27.5
Outpatient department	102	26.3
Pediatrics department	52	13.4
Others^b^	29	7.4
**Had dependent family members**		
Yes	174	44.7
No	215	55.3

^a^Unmarried includes: single (never married), widowed, separated and divorced.

^b^Others refers to ophthalmic, fistula and psychiatry units.

The proportion of nurses who reported they intended to stay in the nursing profession was 39.8%. With respect to job-satisfaction, the majority were satisfied with autonomy and professional opportunities, scheduling, and relationships and interactions (64.5%, 63.5% and 69.9% respectively). On the other hand, most of the respondents were dissatisfied with support, and pay and benefits (55.8% and 67.4% respectively). On the organizational commitment aspect, more than half of the respondents had high continuance and normative commitment (58.9% and 51.9% respectively). On the contrary, majority of the respondents had low affective commitment (51.7%) [Table 3].

**Table 3:** Level of job satisfaction and organizational commitment by different dimensions among nurses in the 5 referral hospitals, Amahara regional state, Ethiopia, 2013

**Variable**	**Number**	**Percent**
**Job-satisfaction**		
Autonomy and professional opportunities		
Unsatisfied	138	35.5
Satisfied	251	64.5
Scheduling		
Unsatisfied	142	36.5
Satisfied	247	63.5
Support		
Unsatisfied	217	55.8
Satisfied	172	44.2
Relationship and interaction		
Unsatisfied	117	30.1
Satisfied	272	69.9
Pay and benefit		
Unsatisfied	262	67.4
Satisfied	127	32.6
**Organizational commitment**		
Affective commitment		
Low	201	51.7
High	188	48.3
Continuance commitment		
Low	160	41.1
High	229	58.9
Normative commitment		
Low	187	48.1
High	202	51.9

### Bivariate and Multivariate Findings: Intent to stay and associated factors

In the bivariate logistic regression analysis; age, marital status, current area of practice, work experience as a nurse, having dependent family members, satisfaction with autonomy and professional opportunities, satisfaction with scheduling, satisfaction with support, satisfaction with relationships and interactions, satisfaction with pay and benefit, having high affective commitment, having high normative commitment and having high continuance commitment showed statistical significance. However, in the multivariate analysis; only age 40–49, marital status, having BSc degree in nursing, satisfaction with autonomy and professional opportunities, satisfaction with schedule, satisfaction with pay and benefit, having high normative commitment and having high continuance commitment were yielded as significantly associated factors of intent to stay in the nursing profession.

Age was significantly associated with intent to stay. Nurses aged 40 to 49 were more likely to stay in their profession than nurses aged 20 to 29 (AOR [95% CI] 4.5 [1.6-12.8]). The other significant factor was marital status with married nurses more likely to report they intended to stay in their profession than those who were unmarried (AOR [95% CI] 2.0 [1.0-3.8]). Nursing education was also among the significant factors as nurses who had bachelor degree in nursing were more likely to report they intended to stay in their profession than nurses who had diploma in nursing (AOR [95% CI] 2.2 [1.2-4.2]).

With respect to job satisfaction, nurses who scored as satisfied on autonomy and professional opportunities were more likely to report they intended to stay in their profession than nurses who scored as unsatisfied (AOR [95% CI] 2.6[1.2-5.9]). Nurses who scored as satisfied on scheduling were more likely to report they intended to stay in their profession than nurses who scored as unsatisfied (AOR [95% CI] 3.4 [1.6-7.5]). Furthermore, nurses who scored as satisfied on pay and benefit were more likely to report they intended to stay in their profession than nurses who scored as unsatisfied (AOR [95% CI] 8.8 [4.5-17.1]).

With respect to organizational commitment, nurses who had high continuance commitment were more likely to report they intended to stay in their profession than nurses who had low continuance commitment (AOR [95% CI] 2.4 [1.3-4.8]). Additionally, nurses who had high normative commitment were more likely to report they intended to stay in their profession than nurses who had low normative commitment (AOR [95% CI] 3.7 [1.8-7.23) [Table 4].

**Table 4:** Bivariate and multivariate logistic regression analysis of factors associated with intent to stay among nurses working in the 5 referral hospitals, Amhara regional state, Ethiopia, 2013

**Variable**	**Intent to stay**		Crud odds ratio with 95% CI*	**Adjusted odds ratio with 95% CI**
	**Yes**	**No**		
Age				
20-29 year	35	81	1.00	1.00
30-39 year	88	133	1.53 (0.95-2.47)	1.74 (0.86-3.44)
40-49 year	32	20	3.70 (1.87-7.35)	4.49 (1.58-12.77)*
Marital status				
Unmarried	38	119	1.00	1.00
Married	117	115	3.19 (2.04-4.98)	1.99 (1.04-3.84)*
Highest level of nursing education				
Diploma	69	119	1.00	1.00
Bachelor degree	86	115	1.29(0.86-1.94)^a^	2.22 (1.18-4.16)**
Current area of practice				
Medical department	34	65	1.00	1.00
Surgical department	37	70	1.01 (0.57-1.80)	0.62 (0.26-1.48)
OPD^b^	40	62	1.23 (0.70-2.20)	0.68 (0.28-1.65)
Pediatric department	28	24	2.23 (1.12-4.43)	1.09 (0.37-3.18)
Others^c^	16	13	2.35 (1.02-5.46)	1.80 (0.44-7.47)
Work experience as a nurse				
0-10 year	113	211	1.00	1.00
11-20 year	34	18	3.53 (1.91-6.53)	1.22 (0.35-4.21)
21-30 year	8	5	2.99 (0.96-9.35)	2.21 (0.20-24.40)
Had dependent family				
Yes	89	85	2.36 (1.56-3.58)	1.12 (0.55-2.28)
No	66	149	1.00	1.00
Autonomy and professional opportunities				
Unsatisfied	14	124	1.00	1.00
Satisfied	141	110	11.35 (6.19-20.82)	2.62 (1.16-5.89)*
Scheduling				
Unsatisfied	17	125	1.00	1.00
Satisfied	138	109	9.31 (5.29-16.39)	3.42 (1.56-7.47)**
Support				
Unsatisfied	43	174	1.00	1.00
Satisfied	112	60	7.55 (4.78-11.94)	1.55 (0.78-3.07)
Relationship and interaction				
Unsatisfied	18	99	1.00	1.00
Satisfied	137	135	5.58 (3.20-9.73)	1.51 (0.70-3.30)
Pay and benefit				
Unsatisfied	48	214	1.00	1.00
Satisfied	107	20	23.85(13.48-42.23)	8.78(4.50-17.14)***
Affective commitment				
Low	48	153	1.00	1.00
High	107	81	4.21 (2.73-6.50)	1.08 (0.55-2.11)
Continuance commitment				
Low	32	128	1.00	1.00
High	123	106	4.64 (2.91-7.40)	2.45 (1.26-4.78)**
Normative commitment				
Low	26	161	1.00	1.00
High	129	73	10.94 (6.61-18.11)	3.68 (1.87-7.23)***

^a^P-value 0.2, ^b^OPD = Outpatient department, ^c^Others include ophthalmic, fistula and psychiatry units, ^*^CI = Confidence Interval.

*P-value <0.05, **P-value <0.001, ***P-value <0.0001.

Hosmer and Lemeshow test: χ^2^ = 4.40, P = 0.82, df = 8.

## Discussion

In this study, the proportion of nurses who intended to stay in the nursing profession was 39.8%. The finding is similar with studies in Hungary (52%) [27] and Taiwan (50.8%) [28]. But it is lower than studies in USA (83.8%) [29] and Ireland (77%) [13] and may be due to differences in job satisfaction as USA and Taiwanese nurses may have higher salaries and more incentives. On the other hand, the proportion is slightly higher than that found in a Korean study (32.8%) [30], which could be attributed to the limited availability of alternative jobs and perceived increased cost that may lead Ethiopian nurses to develop continuance commitment.

With respect to the socio-demographic characteristics, age, marital status and level of nursing education were all associated with Ethiopian nurses’ intent to stay in the profession. Our findings indicated that older nurses (aged 40 to 49 years) were four times more likely to stay in their profession than younger nurses (aged 20 to 29 years); findings consistent with previous research conducted in China [14,15] and South Africa [16]. One possible explanation for this finding might be that older employees have a greater desire for stability or a higher continuance commitment compared to younger nurses due to their family roles. Similarly, married nurses were nearly two times more likely to report they intended to stay in the profession than unmarried ones. This finding is consistent with studies done Yemen, Jordan, Lebanon and Qatar, and Shanghai study [31,32] and might be attributed to the fact that most married nurses in the current study had dependent families, which could create a preference for stability.

Nurses’ level of education was also statistically significant in that nurses who had a bachelor degree in nursing were two times more likely to stay in their profession than nurses who had diploma in nursing. This finding is in contrast to similar studies conducted in Canada [24] and South Africa [16] in which nurses with a diploma were more likely to report intent to stay in their profession than those with a bachelor degree. One possible explanation for this difference might be a contextual difference of the study settings. In the current study setting, there were fewer baccalaureate prepared nurses in the majority of hospitals and most were working as head nurses. Therefore, they may experience greater autonomy and higher job satisfaction compared to the diploma nurses. Moreover, currently nursing education opportunities are relatively better for bachelor nurses than for diploma nurses in the study areas so that bachelor nurses may have a desire to use this opportunity by staying in their profession.

The strongest predictor of intent to stay in the nursing profession in this study was the pay and benefit subscale of job-satisfaction. Nurses who scored as satisfied on pay and benefits were more than eight times more likely to report they intended to stay in their profession than nurses who were unsatisfied. This finding is consistent with previous studies in that job satisfaction has frequently been reported to be the most common predictor for nurses who decided to stay or leave, especially the pay and benefit dimension [12,15,33]. Thus, nurses who perceive that the pay and benefits they receive is proportional to the tasks they accomplish may prefer to stay in their profession rather than leave.

In addition, nurses who had high continuance commitment were two times more likely to stay in their profession than nurses who had low continuance commitment. This finding is also in line with previous studies [15]. Continuance commitment refers to an awareness of the costs associated with leaving the organization [11,20,23]. Therefore, the current finding could most likely be attributed to the nurses’ perception or feeling towards the limited availability of alternative jobs other than the nursing profession or a belief that other jobs may not fulfil their needs as that of nursing.

In the present research, normative commitment was also found as a statistically significant predictor of intent to stay in the nursing profession. Nurses who had high normative commitment were more than three times more likely to report they intended to stay in their profession than nurses who had low normative commitment. Substantial numbers of previous studies support this finding [11,20,34]. Basically normative commitment reflects a feeling of obligation to continue employment. According to previous literatures, the feeling of obligation to remain with an organization may result from familial or cultural socialization which is explained by the internalization of normative pressures exerted on an individual prior to entry into the organization, or it could be the result of organizational socialization that follows entry [20]. However, normative commitment may also develop when an organization provides the employee with prior rewards like paying college fees or covering costs associated with professional training [34], such as the ‘cost-sharing’ policy of Ethiopia in which an agreement is made between the employee and the hospital to pay for the costs of higher education. The other possible explanation might be attributed to the positive feeling of the nurses towards the organization’s visions, missions and values which may lead them to perceive that it is morally correct to stay in their organization as well as in their profession.

The findings of this study also indicated that nurses who were satisfied with autonomy and professional opportunities were two times more likely to report they intended to stay in their profession than those who were unsatisfied. This finding is consistent with other studies in that job satisfaction explained most of the variance in intent to stay [33,35]. For examples, when nurses have greater autonomy for their decisions and better opportunities for professional growth and development, they may have greater job satisfaction and higher organizational commitment that contributes to their desire to stay in their profession.

In this study, the subscale scheduling was also significantly associated with intent to stay. Nurses who scored as satisfied on scheduling were three times more likely to stay in their profession than nurses who scored as unsatisfied. One possible explanation for this finding might be linked with the influence of scheduling on personal and professional development. When nurses get enough time to accomplish their task, they may use their extra time for personal and professional development and so that they may desire to stay in their profession.

Even though, the study covered all nurses working in all referral hospitals found in Amhara regional state it has the following limitations: First, the cross-sectional nature of the study design does not confirm definitive cause and effect relationship. Second, since self-administer questionnaires were used to collect data; the study may be subjected to response bias from each respondent. Finally, the slightly low reliability of affective and continuance commitment subscales suggests the need for item modification in future research.

## Conclusion

Intent to stay in the nursing profession among nurses working in referral hospitals of Amahara regional state is low. The low proportion of nurses who intend to stay in the profession could be a reasonable cause of concern for the regional health bureau and the respective hospital managements to think about the future of the nursing staff. The findings of this study indicate that nurses’ satisfaction with pay and benefits, scheduling, autonomy and professional opportunities, reports of higher normative and continuance commitment, and who have BSc degree in nursing were significantly more likely to report intent to stay in the nursing profession. Therefore, efforts have to be made to increase the professional autonomy of nurses, advance educational opportunities and to make work schedules flexible. Most importantly, revising the current monthly salary and other duty payments of nurses is essential. Future researchers are better to exhaustively address all potential factors associated with intent to stay in the nursing profession with the triangulation of qualitative research methods.

## Abbreviations

AC: Affective Commitment; CC: Continuance Commitment; ITS: Intent to Stay; JS: Job Satisfaction; NC: Normative Commitment; OC: Organizational Commitment.

## Competing interests

The authors declare that they have no competing interests.

## Authors’ contributions

EE, KA and AB participated in all steps of the study from its commencement to write up. They have reviewed and approved the submission of the manuscript. All authors read and approved the final manuscript.

## Pre-publication history

The pre-publication history for this paper can be accessed here:

http://www.biomedcentral.com/1472-6955/13/24/prepub
